# On-target, off-tumor oral toxicities of talquetamab in heavily pretreated multiple myeloma

**DOI:** 10.1007/s00520-026-10583-4

**Published:** 2026-03-20

**Authors:** Fabio Abreu Alves, Ariel Perez Perez, Graziella Chagas Jaguar, Jayr Schmidt Filho, Min Kyeong Kim, Alessandro Villa

**Affiliations:** 1 Stomatology Department, A.C. Camargo Cancer Center, Rua Prof. Antonio Prudente, 21, Liberdade, 01590090, Sao Paulo, SP Brazil; 2 School of Dentistry, Sao Paulo University, Sao Paulo, Brazil; 3 Oral Medicine and Oral Oncology, Miami Cancer Institute, Baptist Health South Florida, Miami, FL USA; 4 Blood and Marrow Transplant Program, Miami Cancer Institute, Miami, FL USA; 5 Department of Surgical Sciences, Herbert Wertheim College of Medicine, Florida International University, Miami, FL USA; 6 Department of Hematology and Cell Therapy, A.C.Camargo Cancer Center, Sao Paulo, Brazil

**Keywords:** Oral toxicities, Talquetamab, Xerostomia, Dysgeusia, Dysphagia

## Abstract

**Background:**

Talquetamab (TAL), a first-in-class bispecific antibody targeting GPRC5D and CD3, has demonstrated high efficacy in heavily pretreated relapsed and refractory multiple myeloma (RRMM), achieving response rates of approximately 70%. However, TAL is frequently associated with on-target, off-tumor oral toxicities.

**Objective:**

This study aimed to review the features of oral toxicities related to TAL.

**Methods:**

A comprehensive review of the recent literature on TAL-associated oral toxicities was conducted. Extracted data included time to onset, incidence, severity grading, and reported management strategies for these adverse events.

**Results:**

Oral effects are hypothesized to arise from immune activation against epithelial cells expressing GPRC5D in the tongue, salivary glands, and hard keratinized tissues. Clinical studies, including the MonumenTAL-1 trial, have reported dysgeusia in up to 72% of patients, dry mouth in 39%, and dysphagia in 24% of patients. Such toxicities typically arise within a few weeks of initiating TAL therapy and tend to persist. They can significantly impair nutrition and quality of life, often leading to weight loss exceeding 6% of baseline body weight. Current management is largely supportive, including corticosteroid mouthwashes, saliva stimulants, nutritional counseling, and TAL dose adjustments, with limited efficacy. The ongoing TALISMAN trial is evaluating prophylactic interventions such as dexamethasone mouthwash and pregabalin.

**Conclusion:**

Oral toxicities often emerge early and may persist throughout the therapy. Consequently, early recognition, preventive care, and multidisciplinary management are essential to mitigate oral complications and preserve quality of life in patients receiving TAL.

## Introduction

The VRd (Velcade—proteasome inhibitor, Revlimid—immunomodulatory agent, dexamethasone—steroid) regimen for multiple myeloma (MM) suppresses malignant plasma cell growth. In the PERSEUS three-phase trial, the addition of daratumumab, an anti-CD38 monoclonal antibody, to VRd during induction and consolidation therapy, along with lenalidomide maintenance therapy, conferred a significant benefit with respect to progression-free survival or death at a median follow-up of 47.5 months. Despite this quadruplet approach and consolidation with autologous stem cell transplantation, relapse may still occur even after an initial response [1–3].

Relapsed and refractory multiple myeloma (RRMM) is defined by the International Myeloma Working Group [3] as disease progression within 60 days of the last therapy after at least a minimal response, or resistance to primary or salvage treatment. Triple-class refractory MM involves resistance to a proteasome inhibitor, an immunomodulatory drug, and an anti-CD38 monoclonal antibody, whereas penta-refractory disease includes resistance to two agents from each of the first two classes and one anti-CD38 antibody. Although CAR T-cell therapies have shown substantial efficacy in heavily pretreated patients, relapse remains frequent, underscoring the need for new treatments [4, 5]. In this context, talquetamab (TAL), a first-in-class bispecific antibody targeting GPRC5D and CD3, has demonstrated significant clinical activity in RRMM and received regulatory approval in 2023 [6, 7]. A 2022 phase I [6] trial demonstrated that TAL was active and tolerable in heavily pretreated MM, establishing the optimal subcutaneous dosing (0.4 mg/kg weekly or 0.8 mg/kg every 2 weeks). The later phase 1–2 study [8] provided a larger patient population and longer follow-up, showing evidence of durable responses (median time, approximately 11 months) and a consistent overall response rate of around 70% across both subgroups, including those previously exposed to T-cell redirection therapy. However, TAL is consistently associated with a high incidence of on-target, off-tumor oral toxicities, most notably dysgeusia, dry mouth, and dysphagia. The clinical presentation includes disturbances of taste (dysgeusia/ageusia), oral dryness, difficulty swallowing, and secondary weight loss. This study aims to provide a concise literature overview of the oral adverse events observed in patients with MM undergoing TAL therapy and discuss their presentation, severity, and practical strategies for prevention and management.

## Methods

A critical search was performed in the MEDLINE/PubMed database. All English-language studies related to oral toxicities associated with TAL were included. The search strategy was based on a combination of the following keywords: (“Talquetamab” OR “GPRC5D”) AND (“oral toxicities” OR “oral side effects” OR “dry mouth” OR “xerostomia” OR “dysgeusia” OR “dysphagia”). Only studies published from 2022 onward were considered (First TAL study- MonumenTAL-1 [6]).

## Results

A total of 25 studies were identified in the literature review. After removal of 11 duplicate studies and 6 review articles reporting data from the MonumenTAL-1 and MonumenTAL-2 trials, 8 studies remained for analysis. These comprised 3 clinical trials [6, 8, 9], 1 prospective study [10], 1 retrospective study [11], 1 case series [12], and 2 case reports [13, 14] (Table 1).

**Table 1:** Clinical presentation and incidence of oral toxicities related to TAL

Study/regimen	Dysgeusia/ageusia	Xerostomia	Dysphagia	Time-to-onset	Resolution/course	Discontinuation due to oral AE
MonumenTAL-1 [6]—0.4 mg/kg weekly	72% of the patients	27%	24% of the patients (0.9% G3/4)	Dysg 10–14 day; X ~ 3rd week; Dysph ~ 6 weeks	Subset of patients without returns to baseline. Dysg duration= 95 days (median). Dysph duration= 109 days (median).	No
MonumenTAL-1 [6]—0.8 mg/kg every other week	71% of the patients	39%	24% of the patients	Same temporal pattern	Subset of patients without returns to baseline. Dysg duration= 102 days(median). Dysph duration= 73 days (median)	< 2%
Iida et al. [9]	66.7% (*n* = 10). Grade 1/2	1 patient (6.7%), grade 1/2	20% (*n* = 3) grade 1/2	Dysg = median time 13.5 days	4 out 10 patients with dysgeusia recovered	No
Laheij et al. [10]—total of 8 patients 6 patients (0.4 mg/kg weekly or 0.8 mg/kg every other week) 2 patients (1600 μg/kg every 4 weeks)	All patients; moderate/severe	7 patients—high grade No difference in salivary flow before and after the initiation of TAL	Not evaluated	Dysg was evaluated after 2 months. Xerostomia = no information	Not shown	Not emphasized
Lery et al. [11] total of 14 patients—0.8 mg/kg every other week	50% (*n* = 7)	71% (*n* = 10)	No	No information	No information	No information
Mansilla-Polo et al. [12] (0.8 mg/kg every other week) Case series—5 patients	3 patients	3 patients	No	No information	No information	No
Gross Even-Zohar et al. [13] (0.8 mg/kg on day 21)	1 patient Grade 2	No	No	No information	Stabilized	No
Kayahara et al. [14] (case report)	1 patient	No	No	3 days after the first escalation dose	Yes	No

*TAL* talquetamab, *AE* adverse event, *Dysg* dysgeusia, *X* xerostomia, *Dysph* dysphagia

## Discussion

**Fig. 1:**
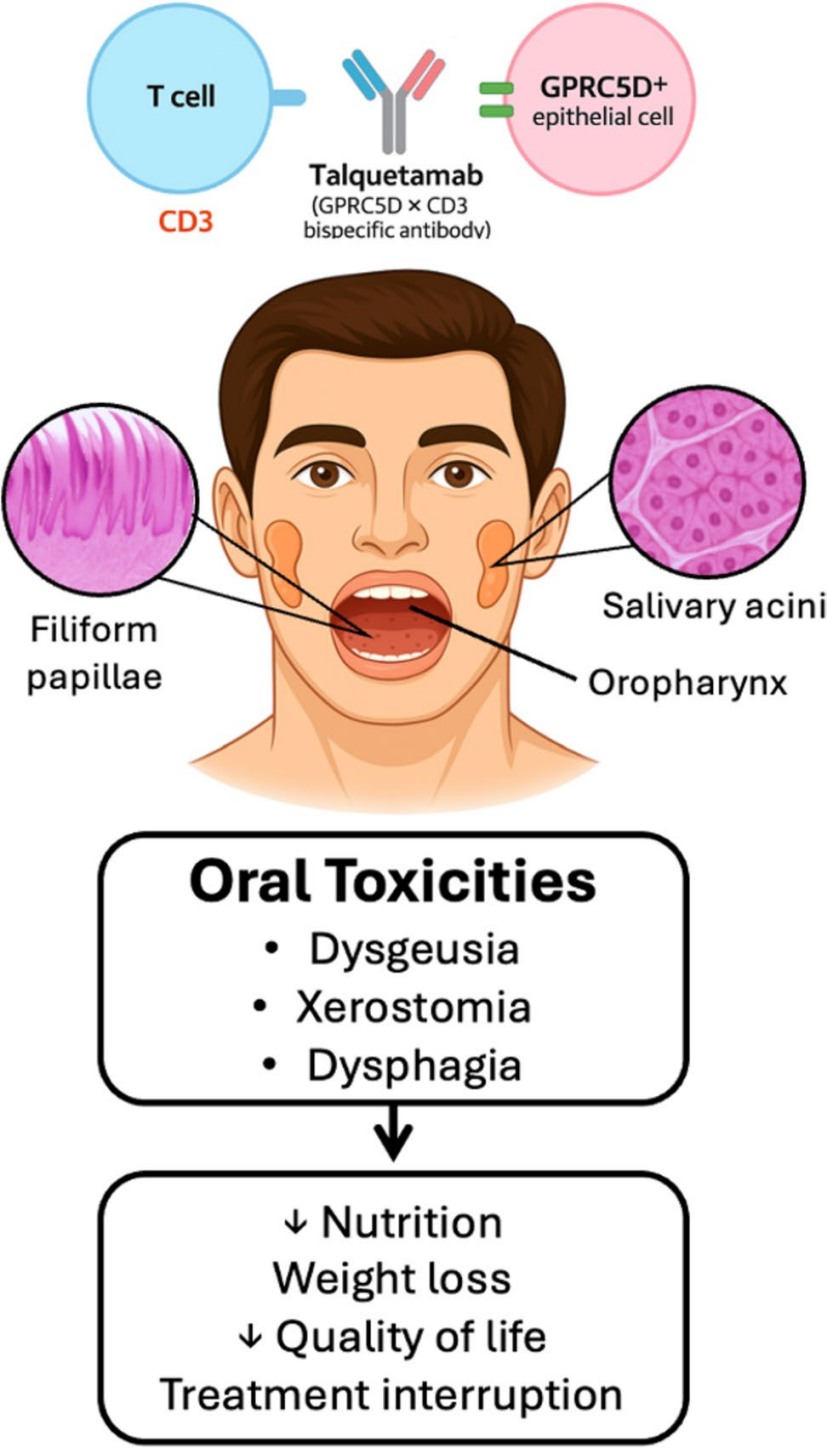
GPRC5D is expressed in the salivary glands and oral tissues, and consequences of the oral toxicities

GPRC5D is an orphan G protein–coupled receptor (GPCR) belonging to family C, group 5 of the metabotropic glutamate receptor–like subfamily. It encodes a seven-transmembrane domain receptor that couples with G proteins to mediate intracellular signaling and is located on chromosome 12p13.3 [15]. Preclinical evidence demonstrates that GPRC5D exhibits pronounced and relatively restricted expression in malignant plasma cells, suggesting a role in plasma cell–specific signaling pathways and supporting its relevance as a therapeutic target in multiple myeloma [16, 17]. Physiologic expression of GPRC5D in normal tissues appears limited and is predominantly associated with hard keratin–producing structures. Inoue et al. [17] demonstrated expression in the keratogenous zone of the nail matrix and in the central regions of the filiform papillae of the tongue through in situ hybridization in murine tissues. In hair follicles, expression was absent during early anagen but became detectable in cortical cells of the developing hair shaft during mid-to-late anagen, persisting until the catagen phase, indicating a role in keratinization and hair shaft formation. Additional immunohistochemical and in situ hybridization analyses presented at the 18th International Myeloma Workshop [18] confirmed GPRC5D expression in epithelial cells of hair follicles and at the base of epithelial columns supporting filiform papillae, with variability in RNA signal likely reflecting differences in follicle density across samples. Data from the Human Protein Atlas [19] further suggest low-level expression in salivary gland tissue, particularly in myoepithelial, ductal, basal, and acinar cells. Collectively, available histologic and transcriptomic evidence indicates that GPRC5D expression in the oral cavity is largely restricted to hard keratinized structures, especially the filiform papillae of the tongue, with limited expression in salivary glands. Biopsies of minor salivary gland showed minimal changes [11]. This localized distribution might support an on-target mechanism for oral adverse events observed with TAL, including dysgeusia—likely related to filiform papillae involvement—xerostomia, possibly associated with salivary gland effects, and mucosal sensitivity attributable to targeting of keratinized epithelium, while explaining the relative sparing of non-keratinized oral mucosa (Fig. 1).

In the MonumenTAL-1 trial [6], dysgeusia was reported in 63% and dry mouth in 30% of patients receiving 0.4 mg/kg weekly, whereas both dysgeusia and dry mouth were observed in 57% of patients in the 0.8 mg/kg every-2-weeks cohort. Dysphagia occurred in 37% and 27% of patients in the weekly and every-2-weeks cohorts, respectively. In the continuation of the multicenter, open-label, phase 1–2 study of MonumenTAL-1 [8], dysgeusia was reported in 72% of patients in the 0.4 mg/kg once-weekly group, and 71% of patients in the 0.8 mg/kg every-2-weeks group, making dysgeusia one of the most frequent adverse events. Dry mouth incidence was approximately 27% in the 0.4 mg/kg weekly group and 39% in the 0.8 mg/kg group, while dysphagia was documented in 24% of patients (grade 3/4 in 0.9%). Symptoms emerged early, with dysgeusia appearing within 10–14 days, dry mouth by the third week, and dysphagia around 6 weeks after treatment initiation. Recovery was often slow, and many patients did not fully return to their baseline. Treatment discontinuation due to these oral toxicities was rare (less than 2% of patients). Across both protocols (0.4 mg/kg vs 0.8 mg/kg), events were mostly of grade 1–2, consistent with a pharmacologic rather than host-level risk signal (Table 1).

Laheij et al. [10] evaluated salivary flow, taste, dry mouth, and quality of life in 8 MM patients before and 8 weeks after initiating TAL. Taste testing demonstrated a significant decline in total taste scores, which was associated with a notable reduction in global health status. Seven of the 8 patients reported severe dry mouth. However, the salivary flow showed no alterations. The Laheij [10] and Chari [6, 8] studies reinforce that these toxicities may represent a mechanism-based class effect mediated by GPRC5D expression in oral epithelial tissues. Taken together, the frequency, persistence, and functional impact of these adverse events support the need for early oral monitoring and timely supportive interventions from the first treatment cycles.

Oncologic patients are at increased risk of malnutrition during treatment, which can lead to sarcopenia, a progressive loss of skeletal muscle mass and strength [20]. This risk is particularly relevant for patients with RRMM, as prior lines of therapy and the disease itself can exacerbate nutritional deficiencies and muscle loss [21]. Therefore, careful assessment and management of nutritional status are essential during TAL treatment. Oral toxicities such as dysgeusia, dry mouth, and dysphagia may further compromise food intake, contributing to weight loss. Moreover, weight loss and appetite loss are closely interconnected and play a central role in the progression of symptom burden during oncologic therapy. Evidence suggests that weight loss not only arises from decreased appetite but also serves as a predictor of subsequent fatigue, emotional distress, and further appetite decline, establishing a self-perpetuating cycle of nutritional and psychological deterioration. As these symptoms often result from treatment-related side effects, as well as inflammatory, metabolic, and oral complications, early recognition and targeted nutritional, psychological, and oral care interventions are essential to preserving patients’ quality of life and maintaining treatment tolerance [20–22].

A 2024 study [22] evaluated 17 patients who received at least one dose of TAL for weight loss and dysgeusia. Among them, 14 patients (82%) experienced significant weight loss (median weight loss, 11.6 lbs (6.1% of baseline weight; range 0–30.4 lbs, 0–14.3%), corresponding to a median BMI decrease of 1.76 (range 0–5.56). One patient developed grade IV weight loss according to the BMI-adjusted Weight Loss Grading System (BMI-WLGS). No correlation was observed between treatment duration and severity of weight loss. Two-thirds of patients developed BMI-WLGS grade ≥ II weight loss accompanied by dysgeusia. On average, patients lost 6% of their initial weight during treatment. Following TAL discontinuation, eight patients regained weight, with five returning to or exceeding their baseline levels. The alteration in taste perception has also been associated with a marked decline in overall health status and quality of life (QoL). Oral health–related QoL also worsened significantly, showing an average increase of 7.7 (± 2.6) points across 11 oral symptoms. This reduction was primarily driven by higher scores in “dry mouth,” “altered taste of food and drinks,” “difficulty eating solid food,” and “mouth sensitivity.” Additionally, dietary modifications resulting from taste changes should be closely monitored by the oncology team, as the relationship between dysgeusia, reduced oral QoL, and weight loss has been well established [10]. A multicenter study evaluating 114 patients showed a mean weight loss of 7.0 kg at 6 months of TAL use, and oral toxicities may be the main contributing factor [23].

Despite the growing recognition of oral toxicities associated with TAL, evidence-based management strategies remain limited, and no prospective studies have specifically evaluated standardized approaches. The ongoing TALISMAN trial (NCT06500884) [24], a phase 2 randomized study, is investigating prophylactic interventions for GPRC5D-related oral toxicities, including dexamethasone mouthwash, pregabalin, and clonazepam; however, results are not yet available (until 02–17−2026). Current knowledge is therefore largely derived from clinical trials [6, 8], investigator experience [25], and real-world practice [26]. Supportive care measures, such as topical corticosteroid or antifungal rinses, zinc or vitamin supplementation, saliva stimulants, hydration, and dietary modifications, have been used to alleviate dysgeusia, although their effectiveness appears variable. Photobiomodulation has shown benefit in isolated cases of TAL-related dysgeusia but requires further validation in controlled studies [14]. However, dose modification has been the most consistently reported strategy for managing severe dysgeusia [25]. Management of xerostomia typically includes saliva substitutes, sugar-free gum or lozenges, and sodium lauryl sulfate–free toothpaste [25], while dysphagia may require oral rinses, analgesics, antifungal therapy, and nutritional support. Prophylactic approaches described in clinical practice include dexamethasone and nystatin mouthwashes, zinc and vitamin B in complex supplementation, and local cooling measures to reduce oral circulation [25]. Furthermore, multidisciplinary care involving dental or oral medicine specialists, nutritional counseling, and proactive patient education is recommended to improve symptom control and quality of life [26].

Conversely, although TAL-related oral toxicities have been reported in approximately 70% of patients in the MonumenTAL-1 and MonumenTAL-2 trials [6, 8], findings from other GPRC5D-targeted approaches suggest a milder oral safety profile. Both the study by Mailankody et al. [27] and the phase 1 trial conducted by Jin et al. [28] evaluating GPRC5D-targeted CAR T-cell therapy in patients with relapsed or refractory multiple myeloma documented only sporadic and low-grade oral adverse events. In the study by Mailankody et al. [27], dysgeusia was reported by two patients (12%), while only one patient (6%) experienced xerostomia, with no severe or persistent symptoms described. Similarly, in the study of Jin et al. [28], none of the 20 treated patients developed oral toxicities. These discrepancies likely stem from differences in drug structure, mechanism of action, dosing schedules, and tissue distribution between bispecific antibodies such as TAL and cellular therapies targeting the same antigen. TAL’s continuous exposure and on-target activity in GPRC5D-expressing keratinized tissues may explain the higher incidence of mucosal and salivary alterations observed in clinical trials, whereas CAR T-cell therapies may result in a more transient or limited interaction with oral tissues. This distinction is clinically relevant for risk stratification, patient counseling, and the development of preventive oral care protocols, while also underscoring the need for standardized reporting criteria and prospective studies to clarify the incidence, mechanisms, and long-term implications of oral adverse events associated with emerging immunotherapies for multiple myeloma.

## Conclusion

Talquetamab has demonstrated efficacy in the treatment of RRMM, with high response rates and durable clinical outcomes. However, it is frequently associated with dysgeusia, taste loss, and dysphagia, which can significantly impair nutrition and quality of life and ultimately compromise treatment adherence. As these toxicities often emerge early and may persist throughout therapy, effective preventive and management strategies should incorporate early risk assessment, individualized care planning, patient education, and timely interventions to mitigate severity, preserve oral function, and support uninterrupted cancer therapy.

## Data Availability

No datasets were generated or analysed during the current study.
